# Recommended oral sodium bicarbonate administration for urine alkalinization did not affect the concentration of mitomycin-C in non-muscle invasive bladder cancer patients

**DOI:** 10.18632/oncotarget.21755

**Published:** 2017-10-09

**Authors:** Ho Kyung Seo, Sung Han Kim, Kyung-Ohk Ahn, Sang-Jin Lee, Weon Seo Park, Sohee Kim, Sang-Hyun Hwang, Do Hoon Lee, Jae Young Joung, Jinsoo Chung, Jungnam Joo, Kyung-Chae Jeong

**Affiliations:** ^1^ Department of Urology, Center for Prostate Cancer, Hospital, National Cancer Center, Goyang, Gyeonggi-do, Korea; ^2^ Biomarker Branch, Research Institute, National Cancer Center, Goyang, Gyeonggi-do, Korea; ^3^ Translational Research Branch, Research Institute, National Cancer Center, Goyang, Gyeonggi-do, Korea; ^4^ Immunotherapeutics Branch, Research Institute, National Cancer Center, Goyang, Gyeonggi-do, Korea; ^5^ Department of Pathology, Center for Prostate Cancer, Hospital, National Cancer Center, Goyang, Gyeonggi-do, Korea; ^6^ Biometrics Research Branch, Research Institute and Hospital, National Cancer Center, Goyang, Gyeonggi, Korea; ^7^ Department of Laboratory Medicine, Center for Diagnostic Oncology, National Cancer Center, Goyang-si, Korea; ^8^ Department of Laboratory Medicine and Hematologic Malignancy Branch, Research Institute and Hospital of National Cancer Center, Goyang-si, Korea

**Keywords:** administration, intravesical therapy, mitomycin C, neoplasm, urinary bladder

## Abstract

**Objective:**

Sodium bicarbonate has been reported to maximize the efficacy of intravesical instillation of mitomycin-C (IVI-MMC) therapy by urine alkalinization in non-muscle-invasive bladder cancer (NMIBC). This study aimed to analyze the changes in MMC concentration according to urinary pH and evaluate the efficacy of sodium bicarbonate to maintain the concentration of active form of MMC during IVI-MMC.

**Methods:**

We prospectively enrolled 26 patients with NMIBC after transurethral resection of bladder tumor. Patients with very high-risk and low-risk NMIBC were excluded. Urinary creatinine, volume, pH, and concentrations of MMC and its degraded form were measured immediately before and after IVI-MMC. The patients were administered 1.5 g of oral sodium bicarbonate during the preceding evening, in the morning, and immediately before the fourth cycle of the six-cycle IVI-MMC. The correlation between MMC concentration and urinary pH changes was explored with or without oral bicarbonate therapy.

**Results:**

Recurrence without progression to muscle-invasive disease was noted in 4 of 26 patients in a 23.7-month follow-up. The mean urinary pH before and after the therapy increased from 6.03 to 6.50, and 6.46 to 7.24, without or with oral SB therapy, respectively. Despite this increase, the concentration of active form of MMC did not change significantly. No correlation was found between urinary pH and MMC concentration. Urine alkalinization by SB administration did not maintain the high concentration of urinary MMC.

**Conclusions:**

Urine alkalinization by sodium bicarbonate administration for IVI-MMC did not maintain the high concentration of active urinary MMC in NMIBC.

## INTRODUCTION

Approximately 70–80% of bladder cancer (BC) initially present with non-muscle-invasive bladder cancer (NMIBC) confined to the mucosa (Ta, Tis) and submucosa (T1) [1, 2]. Nevertheless of its standard transurethral endoscopic resection of bladder tumor (TURB), 50–70% of NMIBC recurs, and 10–15% progresses to muscle-invasive BC (MIBC) [3]. The adjuvant intravesical instillation (IVI) of immuno-chemotherapeutic agents has been proven for prevention of recurrence and progression of NIMBC for more than two decades [4–6]. Bacillus Calmette-Guerin (BCG) is superior in reducing the rate of recurrences, and in preventing or delaying progression to MIBC [7]. However, the shortage of BCG worldwide and its associated local and systemic adverse effects eventually result in withdrawal of treatment in approximately 30–50% patients in real clinical settings [8, 9].

IVI-mitomycin-C (IVI-MMC) is another most commonly used alternative cytotoxic agent for NMIBC. This treatment has lesser local and systemic adverse effects than IVI-BCG; however, it shows lower efficacy [10]. The lower efficacy of IVI-MMC can be explained by primary MMC resistance and inadequate drug delivery to tumor cells [11–13]. Owing to inadequate MMC delivery to BC cells, bladder tissue uptake of IVI-MMC is linearly related to drug concentration in urine [12], and the instability of MMC is characterized in acidic condition. The improved efficacy of IVI-MMC was reported when urinary pH becomes alkaline (pH > 5.5) [11, 13]. Several studies suggested IVI-MMC with oral intake of sodium bicarbonate (SB) for urine alkalinization [11, 13]. This study aimed to analyze the changes in MMC concentration according to urinary pH and evaluate the efficacy of sodium bicarbonate in maintaining the concentration of active form of MMC during IVI-MMC. Our findings may have potential implications in the management of patients with NMIBC.

## RESULTS

### *In vitro* cytotoxicity of MMC according to pH in BC cell line

We evaluated the cytotoxicity of MMC according to pH *in vitro* in advance. Each different MMC solution in regulated pH (5.0–8.0) was incubated at 37°C for 2 h, and the pH of each solution was then adjusted immediately to 7.4 (Supplementary figure 1). MMC inhibited cell survival by 50% at 0.66 μg/mL and pH 5.0 in the KU19-19 cell line and was much less cytotoxic at pH 7.0 (Table 1). However, when cells were treated with MMC at pH 6.5, no significant change in pH change was observed. Table 1 shows that most IC_50_ values of MMC increased more than twofold at pH 5.0, compared with those of fresh MMC.

**Table 1:** *In vitro* cell cytotoxicity of MMC according to pH in bladder cancer cell line

Cell line	pH 5.0	pH 5.5	pH 6.0	pH 6.5	pH 7.0	pH 7.5	pH 8.0	Fresh
**T24**	2.53 ± 0.42	2.01 ± 0.19	1.98 ± 0.33	1.33 ± 0.10	1.44 ± 0.14	1.47 ± 0.14	1.04 ± 0.09	1.16 ± 0.14
**253J**	3.04 ± 0.58	2.64 ± 0.53	2.32 ± 0.41	0.90 ± 0.12	0.92 ± 0.11	0.94 ± 0.12	0.87 ± 0.12	1.03 ± 0.12
**5637**	2.56 ± 0.96	1.86 ± 0.64	1.37 ± 0.50	1.11 ± 0.35	1.26 ± 0.38	1.06 ± 0.31	0.82 ± 0.29	0.93 ± 0.26
**J82**	3.63 ± 0.67	2.83 ± 0.27	2.15 ± 0.24	2.63 ± 0.25	2.65 ± 0.36	2.15 ± 0.23	1.86 ± 0.16	2.12 ± 0.33
**KU19-19**	0.66 ± 0.08	0.63 ± 0.10	0.46 ± 0.06	0.33 ± 0.04	0.32 ± 0.04	0.37 ± 0.06	0.26 ± 0.02	0.31 ± 0.03
**UM-UC-3**	1.46 ± 0.11	1.06 ± 0.08	1.04 ± 0.12	0.95 ± 0.08	0.89 ± 0.08	0.86 ± 0.11	0.83 ± 0.09	0.79 ± 0.06

Average IC_50_ ± standard error (μg/mL).

### Analysis of degraded MMC in regulated pH buffer solutions

We analyzed the degree of MMC degradation in regulated pH (5.0–8.0) buffer conditions. Under acidic conditions, MMC degraded into demethoxylated MMC, and only half of the active MMC remained after a 2-h incubation at pH 5.0 (Table 2). The degradation velocity had gradually diminished with time, and the half-life of active MMC was estimated at 219 min at pH 6.0.

Table 2Degradation of MMC in accordance with diverse pH conditions and time

**Table:** 

	pH 5.0	pH 6.0	pH 7.0	pH 8.0	Fresh
**active MMC**	47.4 ± 0.3	64.2 ± 0.3	95.4 ± 0.5	100.0 ± 0.0	100.0 ± 0.0
**degraded MMC**	52.6 ± 0.3	35.8 ± 0.3	4.6 ± 0.5	0.0 ± 0.0	0.0 ± 0.0

% after 2 hour incubation

**Table:** 

	0 min.	20 min.	40 min.	60 min.	80 min.	100 min.	120 min.
active MMC	100.0 ± 0.0	90.9 ± 0.1	83.8 ± 0.2	77.8 ± 0.3	72.6 ± 0.3	67.9 ± 0.3	64.2 ± 0.3
degraded MMC	0.0 ± 0.0	9.1 ± 0.1	16.2 ± 0.2	22.2 ± 0.3	27.4 ± 0.3	32.1 ± 0.3	35.8 ± 0.3

% at pH 6.0.

### Patient characteristics and adverse events

We conducted clinical study to maximize the efficacy of IVI-MMC. The 26 enrolled patients’ baseline demographics and their information about the urinary samples are described in Table 3. The pH of the overall 144 (71 with SB and 73 without) urinary samples before IVI-MMC significantly increased from 6.23 to 6.87 of 143 (70 with SB and 73 without) samples after IVI-MMC (p<0.001). The median concentrations of urinary MMC and degraded MMC after IVI-MMC were 296.7μg/mL and 22.1μg/mL, respectively. The mean 7% of MMC was degraded after IVI-MMC.

**Table 3:** Characteristics of the study population (N=26)

	Mean ± SD
Age (years)	69.5 (53-84)
Male/Female(%)	22 (84.6)/ 4 (15.4)
Pathological stage	
Ta	17 (65.4)
T1	9 (34.60)
Tumor grade	
G1	3 (11.5)
G2	23 (88.5)
Tumor grade	
low	15 (57.7)
high	11 (42.3)
Concurrent CIS	0
Multiplicity	
Solitary	3 (11.5)
Multiple	23 (88.5)
Repeat TUR-BT	11 (42.3)
T0/Ta	9 (81.8)/ 2 (18.2)
MMC instilled cycle : 6 cycle	22 (85.7)
5 cycle	1 (4.3)
Urinary pH before MMC instillation	6.23 ± 0.72
after MMC instillation	6.87 ± 0.66
Urinary creatinine before MMC instillation	151.6 ± 95.0
Concentration of urinary MMC (ug/mL)	296.7 (84-1324)
Concentration of degraded MMC (ug/mL)	22.1 (3.5-284.3)
Degraded MMC/active MMC	7% (3%-48%)
Follow-up (months)	23.7 ± 7.3
Recurrence	4/26
Progression	0/26

Among the 25 and 49 adverse effects without and with oral SB reported after IVI-MMC, no grade 3 adverse effects affected the IVI-MMC schedule; except for two urinary tract infections which delayed the schedule. (Supplementary Table 1).

### Changes in urinary pH before and after IVI-MMC with or without oral intake of sodium bicarbonate

Oral SB increased the overall pH for both before (6.03 to 6.50) and after MMC (6.46 to 7.24) (p = 0.0085, p < 0.001, respectively Table 4). The MMC concentration in urine was not significantly changed in accordance with administration of SB (p = 0.606); however, the degraded MMC concentration marginally decreased (p = 0.063). Despite not having administered SB pretreatment, only 6 (4.1%) cases had urinary pH ≤ 5.5 after IVI-MMC. No case had urinary pH ≤ 5.5 after IVI-MMC in patients who underwent SB pretreatment (Table 4).

**Table 4:** The change of urinary pH and MMC concentration with or without sodium bicarbonate medication

	without sodium bicarbonate administration (N=23)	with sodium bicarbonate administration (N=23)	P value
Before IVI-MMC	6.03 ± 0.41	6.46 ± 0.60	0.008^a^
After IVI-MMC	6.50 ± 0.48	7.24 ± 0.36	<0.001^a^
Differences of urinary pH before and after IVI-MMC	0.46 ± 0.42	0.78 ± 0.51	0.025^a^
Number of samples/patients before IVI-MMC			
pH ≤5.0	1/1	0/0	
5.0 <pH ≤5.5	13/9	8/5	
5.5 <pH ≤6.0	24/18	12/9	
Number of samples/patients after IVI-MMC			
pH ≤5.0	0/0	0/0	
5.0 <pH ≤5.5	6/5	0/0	
5.5 <pH ≤6.0	13/9	1/1	
MMC concentration (ug/mL)	285.3 (149.3-604.0)	305.3 (129.3-845.3)	0.6057^b^
Degraded MMC concentration (ug/mL)	29.3 (13.3-101.1)	18.9 (8.4-101.1)	0.0634
Degraded MMC/active MMC	0.10 (0.05-0.19)	0.06 (0.04-0.15)	0.0005^b^

^a^Student's t-test, ^b^Wilcoxon's rank sum test; IVI-MMC, intravesical MMC instillation.

### Correlation of MMC concentration with urinary pH and urine volume before and after IVI-MMC

The concentration of MMC was not correlated with urinary pH pre and post IVI-MMC (r = -0.122 and r = 0.053, p > 0.05); however, it was correlated with urinary creatinine (r = 0.630, p < 0.001) and urine volume (r = -0.203, p = 0.017) (Table 5). The concentration of degraded MMC was negatively correlated with urinary pH pre and post IVI-MMC (r = -0.384 and r = -0.346; p < 0.001) and urine volume (r = -0.295, p < 0.001), and positively correlated with urinary creatinine (r = 0.475, p < 0.001).

**Table 5:** Correlation between concentration of MMC and urinary pH, creatinine or urine volume

	concentration of MMC	p-value	concentration of degraded MMC	p-value
Pre MMC urinary pH	-0.1223	0.1545	-0.3836	<0.001
Post MMC urinary pH	0.0527	0.541	-0.3464	<0.001
Urinary Creatinine	0.6300	<0.001	0.4751	<0.001
Urine Volume	-0.2036	0.017	-0.2954	<0.001

Spearman's correlation.

## DISCUSSION

Intravesical chemotherapy has been shown to reduce the rate of NMIBC recurrence, and MMC has become the most commonly used intravesical cytotoxic agent. Despite the popularity of this agent in the treatment of NMIBC, many questions regarding the optimal approach to MMC therapy remain unanswered. The drug concentration rather than dosage determine proportionally the cytotoxicity of intravesical therapies in NMIBC. The high MMC concentration in urine explains the high efficacy rates of cytotoxicity as bladder tissue uptake of MMC is associated with MMC concentration in urine [12]. Previous study also showed improved recurrence-free survival rate and prolonged median time to recurrence with improved MMC activity by the MMC concentration [6].

Drug concentration in urine is dependent on dose, urine production, residual urine volume during instillation, and drug degradation rate [12]. The uptake of the drug into bladder tissue is also dependent on drug properties (molecular weight, hydrophilicity, and lipophilicity), intravesical drug indwelling time, and urothelial integrity [11]. Several methodological studies have been conducted to improve the efficacy of MMC cytotoxicity by using higher dose of instillation concentration, modifying instillation time and urine alkalinization, and using devices to achieve better cell permeability, such as hyperthermia and electrostimulation [10, 11, 17–23]. In a real clinical setting, urothelial integrity after TURB and drug properties could not be controlled in IVI. However, residual urine volume, indwelling time, volume of MMC solution, restriction of fluid intake before IVI-MMC, and IVI-MMC cycles were controllable with a recommendation from BC guidelines [24–26].

Urinary pH could alter the treatment outcome through MMC stability [27]. We evaluated the cytotoxicity of MMC with BC cell lines according to pH condition *in vitro* in advance. Urinary pH was an important factor in increasing MMC cytotoxicity, with a 2× higher increase at pH 5.0–7.0 (Table 1). Based on this result, MMC doses at urinary pH 5.0 should be doubled to optimize the same cytotoxic effect at pH 7.0; similar to previous studies showing SB an effective therapy for preventing inactivation of MMC cytotoxicity [12, 14, 22, 23] and for decreasing risk of tumor recurrence with urine alkalinization (urinary pH > 5.5) [13].

We conducted clinical study to maximize the efficacy of IVI-MMC. In this study, the results showed that urinary pH significantly increased with a median of 0.5 with IVI-MMC, and with a median of 0.47–0.77 with/without SB (p < 0.05, Table 3). However, only 4.1% of the selected patients with urinary pH < 5.0 were worth and agreeable to induce urine alkalinization with SB. Otherwise, the rest should be focused on cell permeability. Also the degradation of MMC was affected by urinary creatinine *in vitro* and *in vivo* study.

The current study focused on the changes in the concentrations of MMC and its degraded form. MMC concentration was not affected by urinary pH as well as the urinary pH increased even after IVI-MMC itself in the real clinical setting (Table 3). Witjes et al. proposed several measures to increase MMC concentration in urine [3]. Another randomized phase III trial similar to this study composed of patients with high-risk NMIBC administered 20 mg or 40 mg IVI-MMC. They alkalinized urine with 1.3 g of oral SB administered the night before, the morning of, and 30 min before intravesical therapy to protect MMC degradation in acidic urine condition. A higher 5-year recurrence-free survival rate than the standard treatment group (42.6% versus 23.5%; p = 0.005) and a longer median time to recurrence (29.1 months versus 11.8 months) were observed in the optimized treatment group [11]. However, the major limitation of this study is the difference in the dose used between treatment groups, which could explain the improved outcomes in the optimized-treatment cohort, regardless of the urine alkalization.

Some limitations in this study include the maintaining dose of SB in the blood during instillation was not provided at each visit, and other influential factors of MMC permeability and cytotoxicity, such as hyperthermic temperature, were not controlled, except for pH in urine. Oncologic outcomes, including recurrence-free survival and progression-free survival, could not be compared because of the crossover design of the study. However this study design is effective to evaluate the concentration of urinary MMC in accordance with urinary pH changes after the administration and efficacious to control a lot of confounding factor in NMIBC. A well-controlled prospective design for simultaneous *in vivo* and *in vitro* studies is significant clinically and scientifically in NMIBC research. In this study, we found that MMC concentration was not affected by urine alkalinization using SB. This phenomenon could be attributed to the increasing effect of urinary pH after IVI-MMC and approximately less than 10% degradation of MMC in urine.

In conclusion,the present recommendation of oral SB for urine alkalinization did not affect the concentration of MMC in most cases. It is necessary to determine which patients with NMIBC do not need oral SB intake, in future studies.

## MATERIALS AND METHODS

### Cell lines and cell viability assay

To determine the effect of MMC on human BC cells (T24, 253J, 5637, J82, KU19-19, and UM-UC-3) supplemented with 10% fetal bovine serum and 1% penicillin/streptomycin solution (Invitrogen, Carlsbad, CA), cells were exposed to each pre-incubated MMC for 2 h and then incubated for further 72 h for the measurement of cell viability using an ATP-based cell viability detection kit (CellTiter-Glo; Promega, Madison, WI). All experiments were performed in quadruple.

### Ethics statements

After approval of the Institutional Review Board of the Research Institution (IRB No. NCCCTS13710) with all patients’ written informed consent, this prospective, single institutional, single-arm, cross-over, comparative study was strictly conducted in accordance with the tenets of the Declaration of Helsinki. The same set of patients were enrolled in with and without oral intake of SB groups, to control several interpersonal patient confounding factors.

### Patient selection and assessment of diagnosing NMIBC

Patients with initially diagnosed NMIBC after TURB were recruited between January 2014 and July 2015. The inclusion criteria were age > 20 years, ECOG performance status 0 to 1, and life expectancy ≥ 24 months. The exclusion criteria were as follows: withdrawal from the study during therapy, failure to follow up 6-week after instillation therapy, stopped taking SB orally, MIBC, urothelial carcinoma of the prostatic urethra, non-urothelial carcinoma of the bladder, and either very high-risk (T1G3 or CIS) and low-risk (primary, solitary, TaG1, <3 cm, and papillary) NMIBC, allergy history to MMC, or small bladder capacity (< 100 cc).

Complete TURB and repeat TURB in T1 or high-grade NMIBC were performed by a single urologist (HKS) prior to IVI-MMC. All tissues were histologically confirmed using the World Health Organization's grading system [14] and the American Joint Committee on Cancer tumor-node-metastasis cancer staging system [15] by a 20-year experienced uropathologist (WSP).

### Study protocol

Weekly 40 mg of 2-hour bladder maintained IVI-MMC (Kyowa Hakko Kogyo Co., Tokyo, Japan) for 6 weeks as induction therapy began 2 to 4 weeks after TURB or repeat TURB. The patients were administered 1.5 g of oral SB at the preceding evening, in the morning, and immediately before the fourth cycle of the IVI-MMC. The urinary sample was collected immediately before and after IVI-MMC. The urinary pH using a pH meter, the concentration of urinary creatinine using a creatinine colorimetric assay kit (Cayman Chemistry, Michigan, USA) and the concentrations of active and degraded forms of MMC using LC-MS/MS were determined immediately before and after IVI-MMC. Adverse effects observed during IVI-MMC on the day of instillation and next visit were classified as local or systemic, and graded in accordance with the Common Terminology Criteria for Adverse Events (version 4.0) [16].

### Measurement of urinary MMC concentration using LC-MS/MS

Each 10 mL of urine sample was filtered through a 0.45-μm nylon filter and loaded onto two Waters Sep-Pak C18 Plus cartridges in tandem. LC-MS/MS quantification was performed using an AB Sciex QTrap® 5500 tandem mass spectrometer (Applied Biosystems, Foster City, CA) in positive electrospray ionization mode. All urine samples were analyzed in duplicate. Quantification was performed using multiple reaction monitoring (MRM) mode. Data acquisition and peak integration were carried out with the AB Sciex Analyst® software.

### Statistical analysis

**Figure 1:**
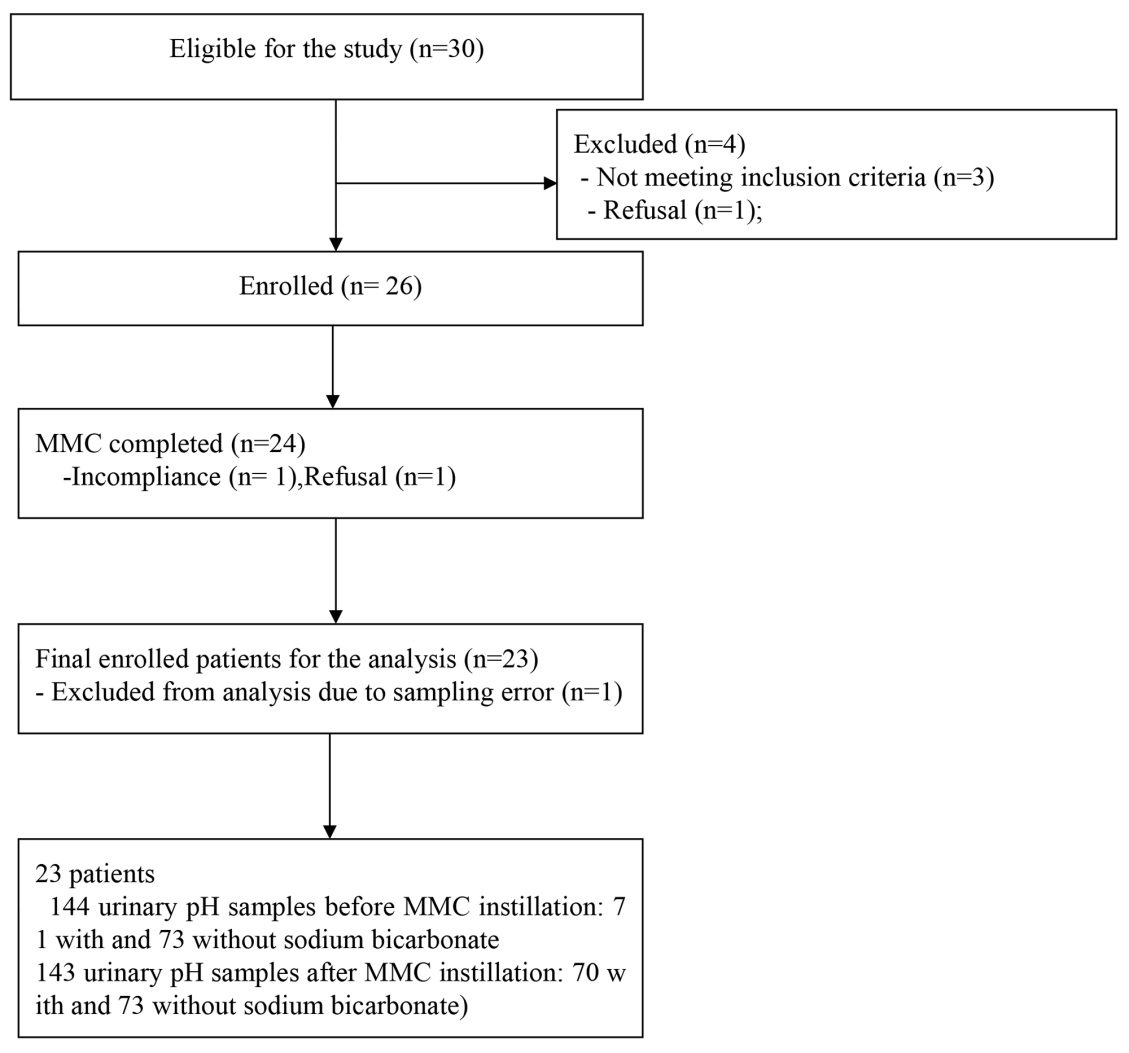
Flow chart of patient enrollment and analysis

The 26 cases were subjected to sample size calculation (http://www.statstodo.com/SSizPairedDiff_Tab.php) by a medical statistician (SK and JJ). To detect the urinary pH, MMC concentration in the pretreated urine samples and the urinary pH was referenced with alpha = 0.05 (2-sided), 90% standard deviation of power within patients = 0.7, and difference in means = 0.5. The calculated number of required samples was 23 cases with 3 additional cases considering the 10% dropout rate, resulting in a final number of 26 cases. Among the thirty patients screened for inclusion in this study; only 23 patients were finally enrolled with 144 urinary samples after the exclusion of 7 patients (Figure 1).

The chi-square test and Fisher's exact test were used to compare the clinical characteristics between the patients who received oral SB with those who did not. All of the statistical analyses were performed using the Stata software version 11.1 (StataCorp, College Station, TX) with a significance of two-sided p values < 0.05.

## SUPPLEMENTARY MATERIALS FIGURES AND TABLES





## Abbreviations

MMC: mitomycin-C
BC: bladder cancer
NMIBC: non-muscle invasive bladder cancer
BCG: Bacillus Calmitte-Guerin
IVI-MMC: intravesical instillation of MMC
TURB: transurethral resection of bladder tumor
