# Supplementary material for: Recommended oral sodium bicarbonate administration for urine alkalinization did not affect the concentration of mitomycin-C in non-muscle invasive bladder cancer patients
Source: Oncotarget. 2017 Oct 9;8(56):96117–25. doi: 10.18632/oncotarget.21755 (PMC5707085; doi:10.18632/oncotarget.21755)
Supplement: Supplementary file 1 [file oncotarget-08-96117-s001.pdf]

Systematic literature review and clinical validation of circulating microRNAs as diagnostic biomarkers for colorectal cancer

SUPPLEMENTARY MATERIALS

Supplementary Figure 1: *In vitro* cytotoxicity of MMC according to pH in BC cell line. Each different MMC solution in regulated pH (5.0–8.0) was incubated at 37°C for 2 h, and the pH of each solution was then adjusted immediately to 7.4. Incubated bladder cancer cells were exposed to each pre-incubated MMC for 2 h and then incubated for further 72 h for the measurement of cell viability using an ATP-based cell viability detection kit (CellTiter-Glo; Promega, Madison, WI). All experiments were performed in quadruple.

See Supplementary File 1

Supplementary Table 1: Adverse events in patients who received at least one dose of mitomycin-C

Toxicity	MMC without sodium bicarbonate (n=24) Number of patients (%)	MMC with sodium bicarbonate (n=24) Number of patients (%)
Urinary urgency	10 (41.7)	12 (50.0)
Urinary frequency	1 (4.1)	7 (29.2)
Urethral pain	4 (16.7)	7 (29.2)
Dysuria	5 (20.8)	4 (16.7)
nocturia	1 (4.1)	2 (8.3)
UTI	1 (4.1)	1 (4.1)
Hematuria	1 (4.1)	4 (16.7)
Headache	1 (4.1)	0
Dermal rash + itching	0	3 (12.5)
constipation	0	1 (4.1)
dizziness	1 (4.1)	0
incontinence	0	2 (8.3)
LBP	0	1 (4.1)
Dry mouth	0	3 (12.5)
chilling & generalize	0	1 (4.1)
Toxicity-related termination of treatment	0	1 (4.1)
