# Supplementary material for: Recommended oral sodium bicarbonate administration for urine alkalinization did not affect the concentration of mitomycin-C in non-muscle invasive bladder cancer patients
Source: Oncotarget. 2017 Oct 9;8(56):96117–25. doi: 10.18632/oncotarget.21755 (PMC5707085; doi:10.18632/oncotarget.21755)
Supplement: Supplementary file 2 [file oncotarget-08-96117-s002.docx]

**Supplementary Figure 1. *In vitro* cytotoxicity of MMC according to pH in BC cell line**

Each different MMC solution in regulated pH (5.0–8.0) was incubated at 37°C for 2 h, and the pH of each solution was then adjusted immediately to 7.4. Incubated bladder cancer cells were exposed to each pre-incubated MMC for 2 h and then incubated for further 72 h for the measurement of cell viability using an ATP-based cell viability detection kit (CellTiter-Glo; Promega, Madison, WI). All experiments were performed in quadruple.


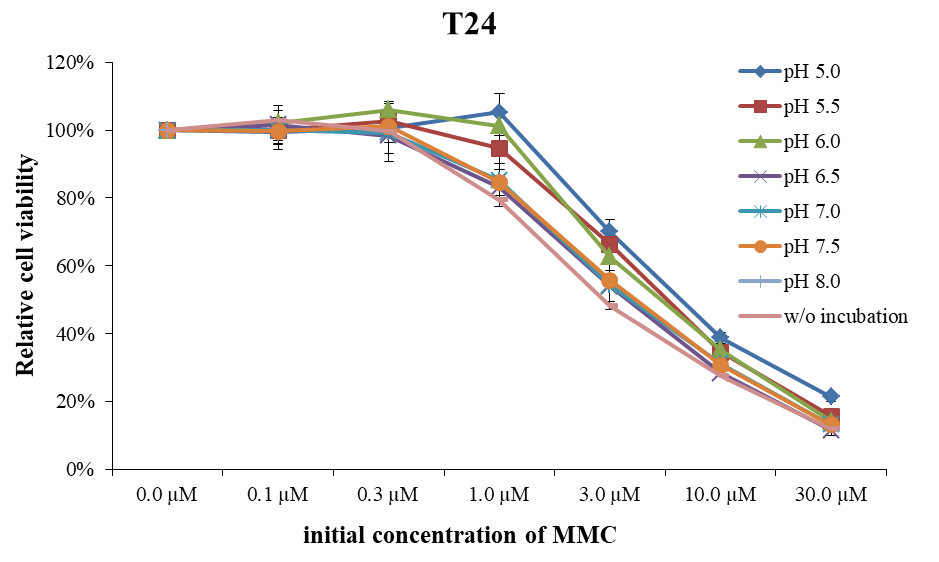


|  |  | **pH 5.0** | **pH 5.5** | **pH 6.0** | **pH 6.5** | **pH 7.0** | **pH 7.5** | **pH 8.0** | **w/o incubation** |
| --- | --- | --- | --- | --- | --- | --- | --- | --- | --- |
| average | 0.0 µM | 1.000 | 1.000 | 1.000 | 1.000 | 1.000 | 1.000 | 1.000 | 1.000 |
|  | 0.1 µM | 0.994 | 0.999 | 1.022 | 1.016 | 1.000 | 0.996 | 0.999 | 1.028 |
|  | 0.3 µM | 1.006 | 1.027 | 1.060 | 0.984 | 0.993 | 1.012 | 0.954 | 0.997 |
|  | 1.0 µM | 1.052 | 0.947 | 1.013 | 0.832 | 0.852 | 0.846 | 0.749 | 0.795 |
|  | 3.0 µM | 0.700 | 0.663 | 0.627 | 0.539 | 0.543 | 0.558 | 0.470 | 0.482 |
|  | 10.0 µM | 0.388 | 0.344 | 0.353 | 0.283 | 0.311 | 0.306 | 0.269 | 0.276 |
|  | 30.0 µM | 0.214 | 0.156 | 0.141 | 0.115 | 0.130 | 0.131 | 0.108 | 0.120 |
| STDEV | 0.0 µM | 0.000 | 0.000 | 0.000 | 0.000 | 0.000 | 0.000 | 0.000 | 0.000 |
|  | 0.1 µM | 0.035 | 0.029 | 0.052 | 0.022 | 0.057 | 0.035 | 0.052 | 0.050 |
|  | 0.3 µM | 0.018 | 0.029 | 0.024 | 0.052 | 0.085 | 0.047 | 0.026 | 0.034 |
|  | 1.0 µM | 0.055 | 0.046 | 0.028 | 0.034 | 0.079 | 0.039 | 0.026 | 0.036 |
|  | 3.0 µM | 0.037 | 0.008 | 0.017 | 0.046 | 0.072 | 0.017 | 0.025 | 0.022 |
|  | 10.0 µM | 0.016 | 0.033 | 0.004 | 0.004 | 0.023 | 0.008 | 0.007 | 0.013 |
|  | 30.0 µM | 0.013 | 0.005 | 0.008 | 0.016 | 0.014 | 0.008 | 0.012 | 0.013 |
| IC50 | (µM) | 7.56 | 6.01 | 5.93 | 3.97 | 4.31 | 4.38 | 3.11 | 3.47 |
|  | (µg/mL) | 2.53 | 2.01 | 1.98 | 1.33 | 1.44 | 1.47 | 1.04 | 1.16 |
| STD ERROR | (µM) | 1.27 | 0.56 | 0.98 | 0.31 | 0.41 | 0.41 | 0.27 | 0.43 |
|  | (µg/mL) | 0.42 | 0.19 | 0.33 | 0.10 | 0.14 | 0.14 | 0.09 | 0.14 |
| R sqr |  | 0.965 | 0.990 | 0.970 | 0.994 | 0.991 | 0.991 | 0.993 | 0.986 |


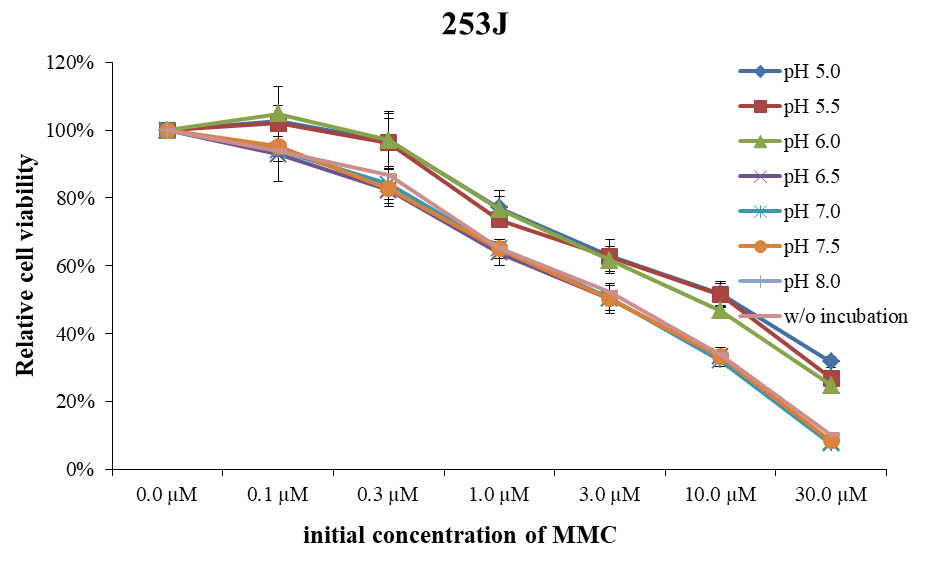


|  |  | **pH 5.0** | **pH 5.5** | **pH 6.0** | **pH 6.5** | **pH 7.0** | **pH 7.5** | **pH 8.0** | **w/o incubation** |
| --- | --- | --- | --- | --- | --- | --- | --- | --- | --- |
| average | 0.0 µM | 1.000 | 1.000 | 1.000 | 1.000 | 1.000 | 1.000 | 1.000 | 1.000 |
|  | 0.1 µM | 1.026 | 1.021 | 1.048 | 0.930 | 0.941 | 0.953 | 0.922 | 0.940 |
|  | 0.3 µM | 0.967 | 0.963 | 0.970 | 0.823 | 0.840 | 0.827 | 0.823 | 0.868 |
|  | 1.0 µM | 0.771 | 0.736 | 0.767 | 0.639 | 0.652 | 0.650 | 0.616 | 0.651 |
|  | 3.0 µM | 0.629 | 0.627 | 0.616 | 0.502 | 0.504 | 0.503 | 0.501 | 0.522 |
|  | 10.0 µM | 0.517 | 0.515 | 0.468 | 0.331 | 0.320 | 0.332 | 0.329 | 0.337 |
|  | 30.0 µM | 0.316 | 0.268 | 0.247 | 0.078 | 0.076 | 0.085 | 0.093 | 0.101 |
| STDEV | 0.0 µM | 0.000 | 0.000 | 0.000 | 0.000 | 0.000 | 0.000 | 0.000 | 0.000 |
|  | 0.1 µM | 0.045 | 0.010 | 0.080 | 0.080 | 0.020 | 0.045 | 0.031 | 0.058 |
|  | 0.3 µM | 0.082 | 0.070 | 0.083 | 0.049 | 0.046 | 0.042 | 0.021 | 0.029 |
|  | 1.0 µM | 0.051 | 0.012 | 0.038 | 0.037 | 0.019 | 0.028 | 0.017 | 0.053 |
|  | 3.0 µM | 0.027 | 0.050 | 0.034 | 0.044 | 0.037 | 0.016 | 0.013 | 0.040 |
|  | 10.0 µM | 0.037 | 0.032 | 0.008 | 0.027 | 0.018 | 0.012 | 0.022 | 0.029 |
|  | 30.0 µM | 0.015 | 0.009 | 0.017 | 0.013 | 0.012 | 0.009 | 0.013 | 0.011 |
| IC50 | (µM) | 9.09 | 7.88 | 6.95 | 2.69 | 2.76 | 2.80 | 2.60 | 3.07 |
|  | (µg/mL) | 3.04 | 2.64 | 2.32 | 0.90 | 0.92 | 0.94 | 0.87 | 1.03 |
| STD ERROR | (µM) | 1.73 | 1.60 | 1.22 | 0.37 | 0.34 | 0.37 | 0.35 | 0.37 |
|  | (µg/mL) | 0.58 | 0.53 | 0.41 | 0.12 | 0.11 | 0.12 | 0.12 | 0.12 |
| R sqr |  | 0.968 | 0.964 | 0.972 | 0.986 | 0.989 | 0.987 | 0.986 | 0.988 |


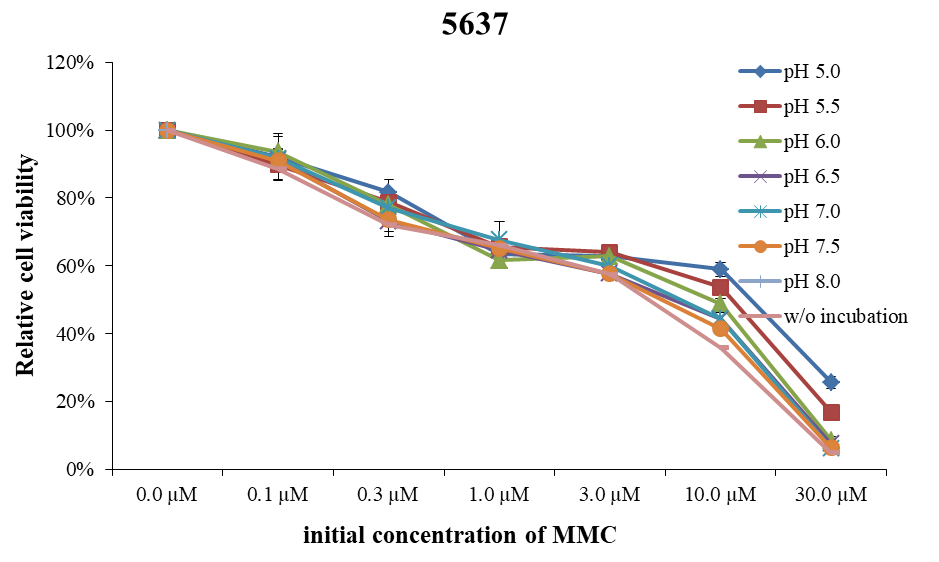


|  |  | **pH 5.0** | **pH 5.5** | **pH 6.0** | **pH 6.5** | **pH 7.0** | **pH 7.5** | **pH 8.0** | **w/o incubation** |
| --- | --- | --- | --- | --- | --- | --- | --- | --- | --- |
| average | 0.0 µM | 1.000 | 1.000 | 1.000 | 1.000 | 1.000 | 1.000 | 1.000 | 1.000 |
|  | 0.1 µM | 0.921 | 0.897 | 0.937 | 0.912 | 0.917 | 0.910 | 0.867 | 0.886 |
|  | 0.3 µM | 0.817 | 0.787 | 0.780 | 0.731 | 0.771 | 0.736 | 0.673 | 0.722 |
|  | 1.0 µM | 0.634 | 0.657 | 0.617 | 0.646 | 0.677 | 0.652 | 0.612 | 0.661 |
|  | 3.0 µM | 0.627 | 0.640 | 0.627 | 0.577 | 0.599 | 0.577 | 0.572 | 0.575 |
|  | 10.0 µM | 0.590 | 0.536 | 0.487 | 0.442 | 0.444 | 0.414 | 0.387 | 0.358 |
|  | 30.0 µM | 0.257 | 0.168 | 0.086 | 0.074 | 0.062 | 0.063 | 0.050 | 0.048 |
| STDEV | 0.0 µM | 0.000 | 0.000 | 0.000 | 0.000 | 0.000 | 0.000 | 0.000 | 0.000 |
|  | 0.1 µM | 0.023 | 0.044 | 0.053 | 0.017 | 0.065 | 0.036 | 0.036 | 0.040 |
|  | 0.3 µM | 0.037 | 0.026 | 0.036 | 0.031 | 0.036 | 0.050 | 0.040 | 0.037 |
|  | 1.0 µM | 0.030 | 0.015 | 0.016 | 0.025 | 0.055 | 0.018 | 0.010 | 0.031 |
|  | 3.0 µM | 0.023 | 0.018 | 0.004 | 0.007 | 0.019 | 0.010 | 0.029 | 0.002 |
|  | 10.0 µM | 0.020 | 0.014 | 0.015 | 0.023 | 0.020 | 0.014 | 0.024 | 0.021 |
|  | 30.0 µM | 0.018 | 0.003 | 0.011 | 0.009 | 0.013 | 0.009 | 0.005 | 0.015 |
| IC50 | (µM) | 7.65 | 5.57 | 4.10 | 3.31 | 3.76 | 3.16 | 2.45 | 2.78 |
|  | (µg/mL) | 2.56 | 1.86 | 1.37 | 1.11 | 1.26 | 1.06 | 0.82 | 0.93 |
| STD ERROR | (µM) | 2.86 | 1.91 | 1.50 | 1.06 | 1.13 | 0.94 | 0.87 | 0.79 |
|  | (µg/mL) | 0.96 | 0.64 | 0.50 | 0.35 | 0.38 | 0.31 | 0.29 | 0.26 |
| R sqr |  | 0.956 | 0.959 | 0.953 | 0.965 | 0.967 | 0.969 | 0.960 | 0.972 |


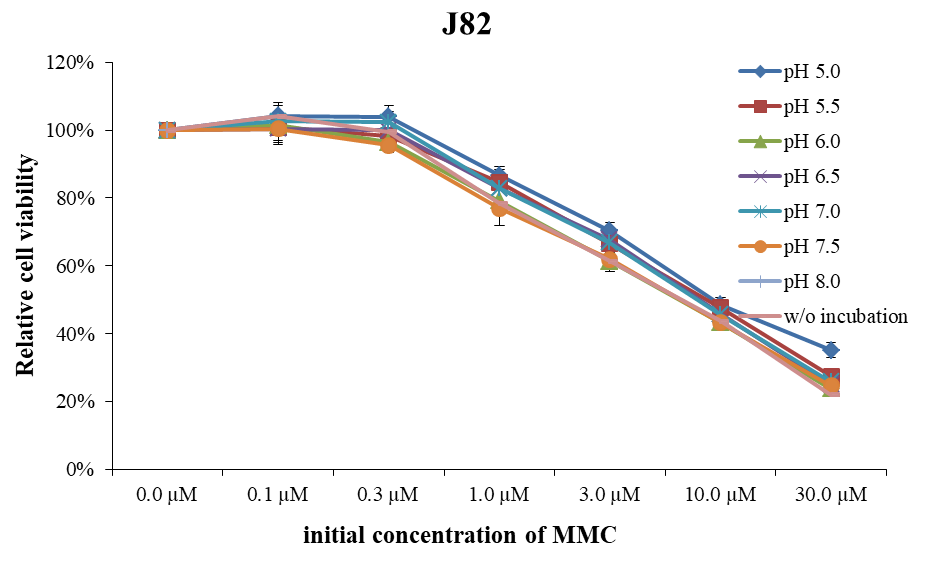


|  |  | **pH 5.0** | **pH 5.5** | **pH 6.0** | **pH 6.5** | **pH 7.0** | **pH 7.5** | **pH 8.0** | **w/o incubation** |
| --- | --- | --- | --- | --- | --- | --- | --- | --- | --- |
| average | 0.0 µM | 1.000 | 1.000 | 1.000 | 1.000 | 1.000 | 1.000 | 1.000 | 1.000 |
|  | 0.1 µM | 1.043 | 1.008 | 1.014 | 1.004 | 1.026 | 1.004 | 0.987 | 1.040 |
|  | 0.3 µM | 1.040 | 0.983 | 0.964 | 1.000 | 1.023 | 0.956 | 0.914 | 0.995 |
|  | 1.0 µM | 0.867 | 0.846 | 0.792 | 0.828 | 0.828 | 0.770 | 0.751 | 0.784 |
|  | 3.0 µM | 0.704 | 0.665 | 0.613 | 0.676 | 0.668 | 0.620 | 0.593 | 0.613 |
|  | 10.0 µM | 0.485 | 0.476 | 0.431 | 0.458 | 0.456 | 0.432 | 0.421 | 0.436 |
|  | 30.0 µM | 0.351 | 0.274 | 0.237 | 0.249 | 0.258 | 0.246 | 0.221 | 0.219 |
| STDEV | 0.0 µM | 0.000 | 0.000 | 0.000 | 0.000 | 0.000 | 0.000 | 0.000 | 0.000 |
|  | 0.1 µM | 0.031 | 0.018 | 0.031 | 0.046 | 0.056 | 0.040 | 0.048 | 0.048 |
|  | 0.3 µM | 0.034 | 0.017 | 0.030 | 0.035 | 0.031 | 0.009 | 0.028 | 0.067 |
|  | 1.0 µM | 0.027 | 0.022 | 0.037 | 0.056 | 0.047 | 0.051 | 0.034 | 0.021 |
|  | 3.0 µM | 0.023 | 0.025 | 0.031 | 0.018 | 0.029 | 0.038 | 0.031 | 0.030 |
|  | 10.0 µM | 0.022 | 0.020 | 0.013 | 0.019 | 0.020 | 0.007 | 0.025 | 0.021 |
|  | 30.0 µM | 0.022 | 0.006 | 0.014 | 0.016 | 0.028 | 0.028 | 0.012 | 0.027 |
| IC50 | (µM) | 10.84 | 8.48 | 6.42 | 7.87 | 7.93 | 6.44 | 5.57 | 6.34 |
|  | (µg/mL) | 3.63 | 2.83 | 2.15 | 2.63 | 2.65 | 2.15 | 1.86 | 2.12 |
| STD ERROR | (µM) | 2.01 | 0.79 | 0.71 | 0.75 | 1.08 | 0.70 | 0.48 | 0.98 |
|  | (µg/mL) | 0.67 | 0.27 | 0.24 | 0.25 | 0.36 | 0.23 | 0.16 | 0.33 |
| R sqr |  | 0.964 | 0.991 | 0.988 | 0.990 | 0.981 | 0.989 | 0.993 | 0.978 |


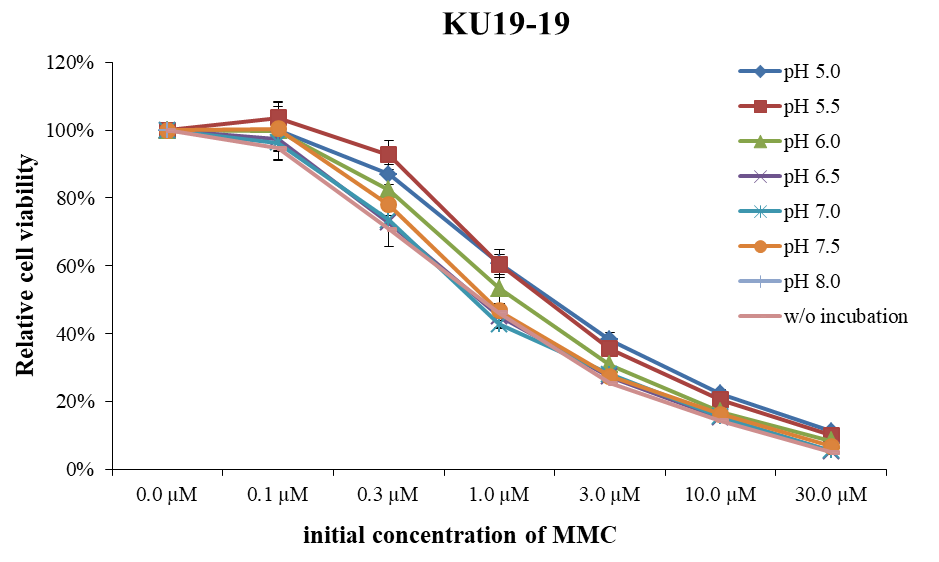


|  |  | **pH 5.0** | **pH 5.5** | **pH 6.0** | **pH 6.5** | **pH 7.0** | **pH 7.5** | **pH 8.0** | **w/o incubation** |
| --- | --- | --- | --- | --- | --- | --- | --- | --- | --- |
| average | 0.0 µM | 1.000 | 1.000 | 1.000 | 1.000 | 1.000 | 1.000 | 1.000 | 1.000 |
|  | 0.1 µM | 1.000 | 1.037 | 0.998 | 0.975 | 0.963 | 1.005 | 0.898 | 0.947 |
|  | 0.3 µM | 0.870 | 0.928 | 0.824 | 0.727 | 0.734 | 0.780 | 0.698 | 0.707 |
|  | 1.0 µM | 0.607 | 0.604 | 0.534 | 0.451 | 0.428 | 0.467 | 0.402 | 0.461 |
|  | 3.0 µM | 0.382 | 0.355 | 0.308 | 0.273 | 0.280 | 0.274 | 0.241 | 0.254 |
|  | 10.0 µM | 0.223 | 0.204 | 0.170 | 0.151 | 0.154 | 0.162 | 0.137 | 0.144 |
|  | 30.0 µM | 0.112 | 0.099 | 0.084 | 0.053 | 0.054 | 0.068 | 0.051 | 0.050 |
| STDEV | 0.0 µM | 0.000 | 0.000 | 0.000 | 0.000 | 0.000 | 0.000 | 0.000 | 0.000 |
|  | 0.1 µM | 0.034 | 0.046 | 0.085 | 0.064 | 0.026 | 0.066 | 0.042 | 0.054 |
|  | 0.3 µM | 0.030 | 0.042 | 0.036 | 0.071 | 0.015 | 0.021 | 0.059 | 0.030 |
|  | 1.0 µM | 0.042 | 0.030 | 0.046 | 0.037 | 0.011 | 0.020 | 0.026 | 0.019 |
|  | 3.0 µM | 0.022 | 0.021 | 0.023 | 0.015 | 0.023 | 0.010 | 0.018 | 0.009 |
|  | 10.0 µM | 0.011 | 0.011 | 0.012 | 0.009 | 0.009 | 0.005 | 0.005 | 0.005 |
|  | 30.0 µM | 0.006 | 0.005 | 0.010 | 0.004 | 0.010 | 0.006 | 0.005 | 0.008 |
| IC50 | (µM) | 1.97 | 1.90 | 1.38 | 0.98 | 0.95 | 1.10 | 0.77 | 0.92 |
|  | (µg/mL) | 0.66 | 0.63 | 0.46 | 0.33 | 0.32 | 0.37 | 0.26 | 0.31 |
| STD ERROR | (µM) | 0.24 | 0.30 | 0.17 | 0.13 | 0.13 | 0.17 | 0.07 | 0.10 |
|  | (µg/mL) | 0.08 | 0.10 | 0.06 | 0.04 | 0.04 | 0.06 | 0.02 | 0.03 |
| R sqr |  | 0.989 | 0.980 | 0.989 | 0.993 | 0.993 | 0.991 | 0.997 | 0.996 |


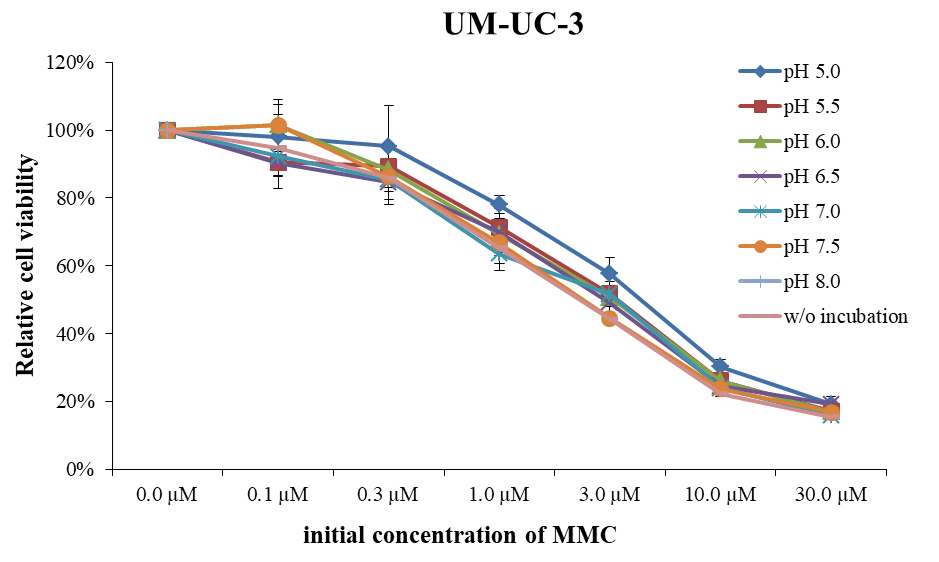


|  |  | **pH 5.0** | **pH 5.5** | **pH 6.0** | **pH 6.5** | **pH 7.0** | **pH 7.5** | **pH 8.0** | **w/o incubation** |
| --- | --- | --- | --- | --- | --- | --- | --- | --- | --- |
| average | 0.0 µM | 1.000 | 1.000 | 1.000 | 1.000 | 1.000 | 1.000 | 1.000 | 1.000 |
|  | 0.1 µM | 0.980 | 0.906 | 1.016 | 0.904 | 0.923 | 1.015 | 0.977 | 0.948 |
|  | 0.3 µM | 0.953 | 0.893 | 0.883 | 0.846 | 0.853 | 0.862 | 0.883 | 0.858 |
|  | 1.0 µM | 0.780 | 0.714 | 0.693 | 0.699 | 0.636 | 0.668 | 0.665 | 0.653 |
|  | 3.0 µM | 0.576 | 0.517 | 0.507 | 0.491 | 0.516 | 0.445 | 0.437 | 0.442 |
|  | 10.0 µM | 0.302 | 0.261 | 0.261 | 0.245 | 0.239 | 0.238 | 0.228 | 0.223 |
|  | 30.0 µM | 0.191 | 0.171 | 0.169 | 0.191 | 0.159 | 0.169 | 0.172 | 0.155 |
| STDEV | 0.0 µM | 0.000 | 0.000 | 0.000 | 0.000 | 0.000 | 0.000 | 0.000 | 0.000 |
|  | 0.1 µM | 0.095 | 0.044 | 0.031 | 0.038 | 0.095 | 0.077 | 0.102 | 0.048 |
|  | 0.3 µM | 0.120 | 0.050 | 0.027 | 0.066 | 0.057 | 0.043 | 0.050 | 0.068 |
|  | 1.0 µM | 0.027 | 0.025 | 0.088 | 0.069 | 0.050 | 0.030 | 0.080 | 0.041 |
|  | 3.0 µM | 0.047 | 0.038 | 0.025 | 0.041 | 0.057 | 0.016 | 0.036 | 0.024 |
|  | 10.0 µM | 0.021 | 0.002 | 0.014 | 0.031 | 0.018 | 0.007 | 0.000 | 0.010 |
|  | 30.0 µM | 0.015 | 0.013 | 0.010 | 0.024 | 0.009 | 0.006 | 0.007 | 0.015 |
| IC50 | (µM) | 4.38 | 3.18 | 3.12 | 2.86 | 2.65 | 2.56 | 2.49 | 2.35 |
|  | (µg/mL) | 1.46 | 1.06 | 1.04 | 0.95 | 0.89 | 0.86 | 0.83 | 0.79 |
| STD ERROR | (µM) | 0.33 | 0.24 | 0.34 | 0.25 | 0.25 | 0.33 | 0.27 | 0.17 |
|  | (µg/mL) | 0.11 | 0.08 | 0.12 | 0.08 | 0.08 | 0.11 | 0.09 | 0.06 |
| R sqr |  | 0.997 | 0.998 | 0.995 | 0.997 | 0.997 | 0.993 | 0.995 | 0.998 |
